# Clinical Features, Treatment, and Prognostic Factors in Neuronal Surface Antibody-Mediated Severe Autoimmune Encephalitis

**DOI:** 10.3389/fimmu.2022.890656

**Published:** 2022-06-02

**Authors:** Baojie Wang, Chunjuan Wang, Jianli Feng, Maolin Hao, Shougang Guo

**Affiliations:** ^1^ Department of Neurology, Shandong Provincial Hospital, Cheeloo College of Medicine, Shandong University, Jinan, China; ^2^ Department of Neurology, Shandong Second Provincial General Hospital, Jinan, China; ^3^ Department of Neurology, Shandong Provincial Hospital, Shandong First Medical University, Jinan, China; ^4^ Department of Neurology, Shandong Provincial Hospital, Shandong University, Jinan, China

**Keywords:** severe, autoimmune encephalitis, rituximab, bortezomib, treatment and prognosis

## Abstract

**Objective:**

This study aimed to determine the clinical characteristics and evaluate the efficacy of immunotherapy and the long-term prognosis of severe autoimmune encephalitis (AE) in China.

**Methods:**

Clinical features, laboratory or radiological findings, and treatment outcomes of 60 severe patients with AE from January 1, 2014, to December 31, 2020, were collected. Continuous variables were compared using the *t*-test and the nonparametric Mann–Whitney *U* test, as appropriate. Univariate and multivariable logistic regression analyses were performed to assess the correlations between factors, treatment responses, and prognosis of severe AE.

**Results:**

The median age of symptom onset was 35 years. Tumors were identified in 23.3% of patients, and 36/60 (60%) patients responded to first-line immunotherapy. Second-line immunotherapy was implemented in 26/60 (43.3%) patients. A significant clinical benefit was observed in 19/26 (73.1%) patients treated with lower dosage rituximab; seven patients were still refractory and received bortezomib as an add-on therapy. During the last follow-up, 48/60 (80%) patients achieved good outcomes (mRS, 0–2), and 10 died. Seventeen patients experienced relapses. A high CD19^+^ B-cell count (OR, 1.197; 95% CI [1.043–1.496]; *p* = 0.041) and a lower neutrophil-to-lymphocyte ratio (NLR; OR, 0.686; 95% CI [0.472–0.884]; *p* = 0.015) predict the response to first-line treatment and good prognosis, respectively.

**Conclusions:**

Patients with severe AE were in critical condition at baseline but could be salvaged after effective rescue immunotherapy. A lower dosage of rituximab could be an optimal option for severe AE. CD19^+^ B-cell count and NLR may provide prognostic information for predicting treatment response and outcome of severe AE.

## Introduction

Autoimmune encephalitis (AE) constitutes a group of diseases with autoantibodies against neurosurface and synaptic antigens, characterized by abnormal psychiatric behavior or cognitive dysfunction, speech dysfunction, seizures, movement disorder, decreased levels of consciousness, autonomic dysfunction, and central hypoventilation (1). Following infectious encephalitis, AE is the second most common cause of encephalitis, with an estimated incidence of approximately 6.5/10,000 (2, 3). Approximately 80%–85% of patients with AE respond favorably to timely immunosuppressive therapies (4); however, a significant portion of patients with AE progress to critical conditions and often require long-term hospitalization.

The pathogenic mechanisms of severe AE remain poorly understood. Previous studies have shown that innate immunity plays a role in AE pathogenesis (5). Neutrophils, monocyte infiltration, and several proinflammatory cytokines produced by neutrophils during neuroinflammatory conditions are known to affect the function of the blood–brain barrier (BBB), leading to increased permeability of immune cells and inflammatory mediators (6). Lymphocytes can permeate through the damaged BBB and differentiate into plasma cells. Dysfunction of the BBB and intrathecal immunopathogenesis by the infiltration of B cells and CD138^+^ antibody-secreting cells are considered responsible for disease severity (7–9). In addition, tumors express a wide variety of nontissue-specific surface proteins, including neuronal antigens that can be presented to T cells, generating an immune response against the central nervous system. Moreover, genetic analysis of paraneoplastic syndrome (PNS)-associated tumors has revealed specific molecular signatures and mutations in genes encoding onconeural proteins, leading to the production of highly immunogenic neoantigens, which may also contribute to disease pathogenesis (10).

Patients may suffer from the poor consequences of severe AE with functional and psychosocial sequelae due to delayed diagnosis and therapy. Hence, the emphasis on timely and effective interventions for severe AE has increased, which may salvage this critical zone and consequently prevent disease progression and relapse, facilitating neurological function recovery. The development of monoclonal antibody treatment and protease inhibitors has made significant progress since the characterization of the targeted depletion of B cells and long-lived plasma cells (9, 11–13).

Current knowledge regarding severe AE is limited, and more detailed information about its epidemiologic and clinical characteristics, the potential mechanisms of severe AE, and more effective regimens for severe AE are needed. In this study, we performed a retrospective cohort analysis of patients with severe AE. The main challenges confronted in clinical practice are discussed, which will contribute to innovations in the exploration of severe AE.

## Methods

### Study Design and Patients

This study was approved by the institutional review board of Shandong Provincial Hospital (SWYX : No.2022-160). All procedures performed on human participants were in accordance with the ethical standards of the institutional committee and with the 1964 Helsinki Declaration. Informed consent was obtained from all participants or their legal representatives.

**Figure 1:**
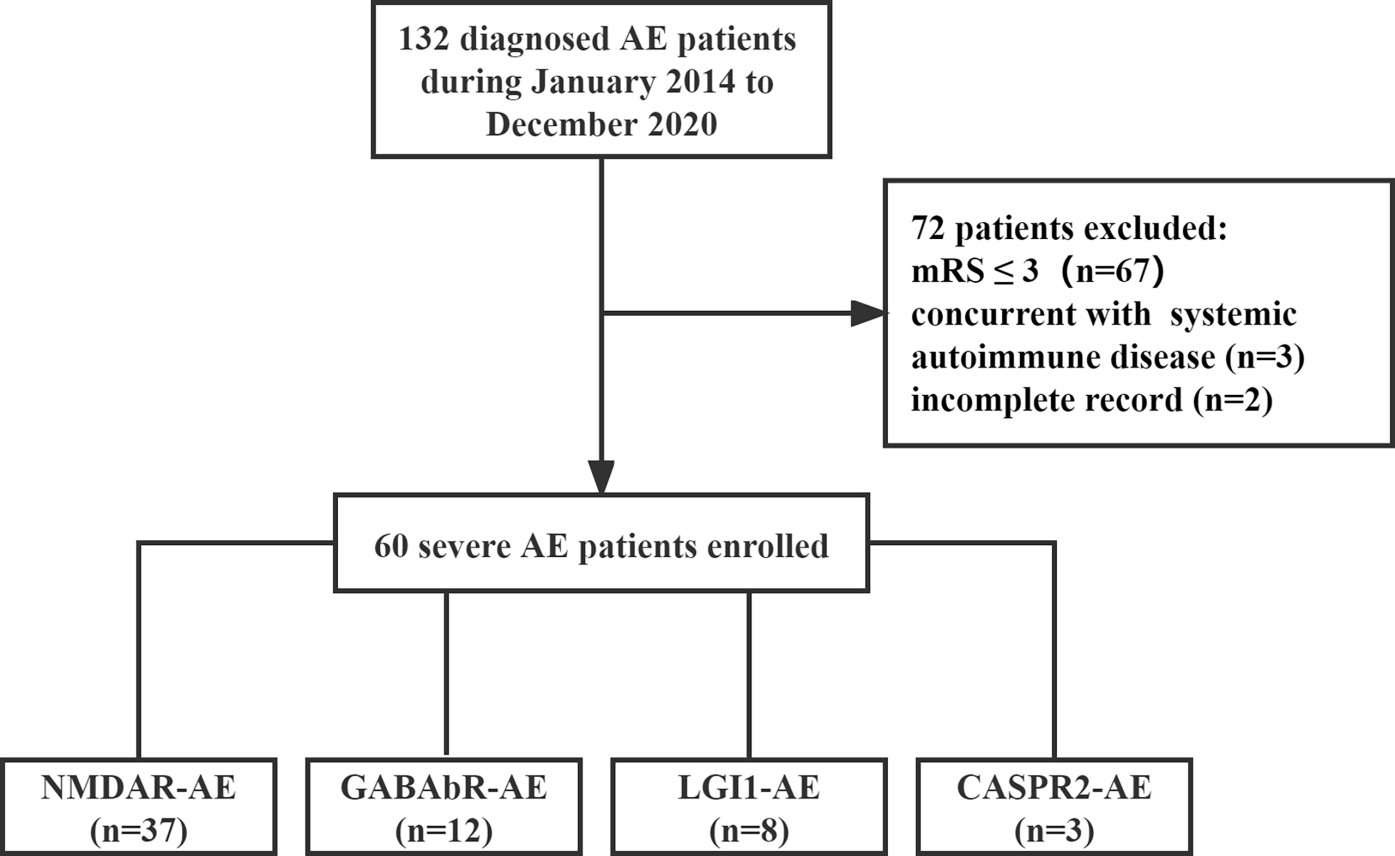
The flowchart of included study.

Patients with severe AE admitted to Shandong Provincial Hospital between January 1, 2014, and December 31, 2020, were enrolled in this retrospective study (**Figure 1**). The inclusion criteria were as follows: (1) the presence of one or more of the following six major groups of symptoms: abnormal psychiatric behavior or cognitive dysfunction; speech dysfunction (pressured speech, verbal reduction, mutism); seizures, movement disorder, dyskinesias, or rigidity/abnormal postures; decreased level of consciousness; autonomic dysfunction; or central hypoventilation. (2) The presence of serum or cerebrospinal fluid (CSF) autoantibodies to neuronal cell surface antigens, including *N*-methyl-d-aspartate receptor (NMDAR), leucine-rich glioma-inactivated 1 (LGI1), contactin-associated protein-like 2 (CASPR2), gamma-aminobutyric acid-b receptor (GABAbR), and α-amino-3-hydroxy-5-methyl-4-isoxazole propionic acid 1/2 (AMPA1/2) receptor, were analyzed using cell-based assays. (3) Severe neurological dysfunction at the onset of disease with a modified Rankin scale (mRS) score of 4–5. (4) Respiratory failure leading to ventilator support. (5) Status epilepticus or decreased consciousness requiring care in the intensive care unit (ICU). Patients with concurrent systemic autoimmune disease and neurological dysfunction at the onset of the disease with a mRS score of 0–3 and incomplete records were also excluded from this study.

Demographic data included sex, age at AE onset, antibody profile, clinical features, and neuroimaging findings. To screen for an associated neoplasm, all patients underwent a CT scan of the thorax/abdomen/pelvis, an ultrasound of the abdomen and the pelvic region, and a transvaginal ultrasound was performed in married women. Peripheral B-cell levels (CD19^+^ B-cell count) and routine blood examinations were performed on freshly acquired blood samples within 12 h of admission before any immunosuppressive treatment. The neutrophil-to-lymphocyte ratio (NLR) is defined as the number of neutrophils divided by the number of lymphocytes and is used to assess the state of inflammation in the body. Patients with infectious diseases were excluded, which may have had a potential impact on white blood cell counts. Lumbar punctures were performed on the second day after admission, and CSF protein and white cell counts were analyzed.

The immunotherapy treatment forms and application time points were reviewed. First-line immunotherapy was defined as corticosteroid therapy at a dosage of 500–1,000 mg for 3–5 days and 0.4 g/kg intravenous immunoglobulins (IVIG) for 5 days. Second-line immunotherapies include rituximab and cyclophosphamide, alone or in combination. Long-term immunotherapy was mycophenolate mofetil (MMF) of >1 year; bortezomib was used as an add-on immunotherapy. Rituximab infusion was administered when there was no meaningful clinical response (improvement in the mRS, <1 point) after 2–4 weeks of optimized first-line therapy or when patients relapsed despite long-term immunotherapy. Bortezomib was administered to rituximab-resistant patients who showed no substantial improvement after the last dose of rituximab for at least 1 month.

Good outcomes or functional independence were defined as mRS 0 to 2; relapse was defined as the appearance of new-onset symptoms or the worsening of preexisting symptoms after improvement or stabilization of the disorder for at least 2 months, not explainable by other causes. Early diagnosis was defined as the median duration from the disease to diagnosis.

### Statistical Analysis

Data were analyzed using R software. Continuous variables are expressed as means ± standard deviations; otherwise, numerical variables are described as medians and ranges. Continuous variables with >2 subgroups were compared using the Kruskal–Wallis test, and two subgroups were compared using the Mann–Whitney *U* test or *t*-test. Factors affecting outcomes were assessed using univariate logistic regression analysis. Clinically or statistically relevant variables from the univariate analyses were used in the multivariate logistic regression analysis. Receiver operating characteristic (ROC) curve analysis was performed to assess the predictive performance for outcomes based on the NLR values and CD19^+^ B-cell count obtained at admission. The cutoff values were estimated using the ROC curve, and the corresponding sensitivities and specificities were calculated based on the area under the curve (AUC). Statistical significance was set at *p* < 0.05.

## Results

### Epidemiological Characteristics

**Figure 2:**
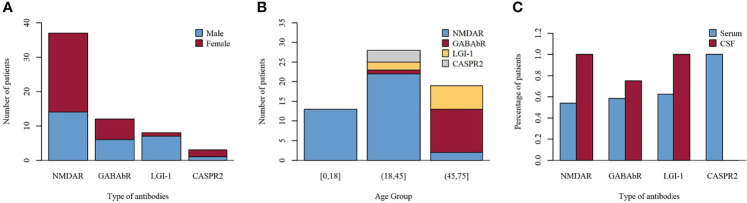
Frequency of distributions of sex **(A)**, age **(B)**, and antibodies **(C)** among subtypes of autoimmune encephalitis.

We investigated the clinical course of 60 severe patients with AE associated with antibodies against neuronal cell surface antigens. The ratio of female to male patients was 8:7. Among all patients, 37 (61.7%) were positive for anti-NMDAR antibodies (23 female and 14 male patients), 12 (20%) for anti-GABAbR antibodies (6 female and 6 male patients), 8 (13.3%) for anti-LGI1 antibodies (1 female and 7 male patients), and 3 (5%) for anti-CASPR2 antibodies (2 female and 1 male patient) (**Figure 2A**). The median age of 60 severe AE was 35 years (range, 14 to 72 years). The percentage of patients under 18 years old, in the age range between 18 and 45 years, and older than 45 years was 13 (21.7%), 30 (50%), and 17 (28.3%), respectively (**Figure 2B**). The sensitivity of antibody testing in the serum and CSF of all the patients with severe AE was 57.6% and 91.5%, respectively. All 37 patients with NMDAR-AE were CSF positive, and 20 (54%) were seropositive. Among the GABAbR-AE cases, seven (58.3%) had detectable antibodies in the serum, and nine (75%) were positive for antibodies in the CSF. For LGI1-AE, antibodies were found in the serum and CSF in five (62.5%) and eight (100%) cases, respectively. All three CASPR2-positive patients had anti-CASPR2 antibodies in serum (**Figure 2C**).

### Clinical Characteristics

The median time lag from symptom onset to diagnosis of severe AE was 19 days (ranging from 3 to 180 days), and the clinical manifestation of severe AE displayed a distinct phenotype. In the initial description, the most common clinical symptoms were seizures (43 patients, 73%), psychosis (23 patients, 39%), and decreased level of consciousness (21 patients, 36%). In our study, we noted that psychosis (23 patients, 62.2%) was most frequent in the NMDAR subgroup. Seizures occurred in the 12 GABAbR-positive patients; eight patients with LGI1 antibodies presented with seizures or cognitive impairment, which was in line with previously reported studies. The median mRS at the onset of the disease was 5 (range, 4 to 5), and 49 patients (81.7%) had an mRS score of 5. Twenty-four patients (40.6%) were admitted to the ICU because of status epilepticus, central hypoventilation requiring respiratory support, and serious complications.

Associated tumors were detected in 14 patients (23.3%). Five ovarian teratomas (13.5%) were identified in patients with NMDAR encephalitis (median age, 22 years), and complete tumor resection was performed at a median time of 16.5 days after disease onset. Two patients showed neurologic improvement, and the other three did not respond. In the GABAbR-AE group, 9 (75%) patients were diagnosed with small-cell lung cancer (median age of 67 years). Seven of the 9 patients with a tumor were treated with surgery or chemotherapy; however, only 3 patients showed a partial response. Compared to the NMDAR-AE and GABAbR-AE groups, patients with LGI1-AE and CASPR2-AE did not show the presence of underlying cancer on tumor screening.

### Auxiliary Examinations

T2 fluid-attenuated inversion recovery showed high signal in the bilateral temporal lobes (6 patients, 10%), hippocampus (7 patients, 11.7%), parietal lobe (3 patients, 5%), and cortex (2 patients, 33.3%). The CSF analysis revealed lymphocytic pleocytosis (1–286 cells/L; median, 10 cells/L) in 25 (41.7%) patients, while 11 (18.3%) patients had increased protein concentration (0.1–1.39 g/L; median, 0.3 g/L). Inflammatory changes in CSF most frequently occur in the NMDAR-AE and GABAbR-AE groups. The distribution of CSF white cell counts and protein concentrations in the different AE subtypes is shown in **Table 1**.

**Table 1:** Characterization of the whole cohort.

	Total cases	NMDAR	GABAbR	LGI1	CASPR2
*N*	60	37	12	8	3
Female/male (*n*)	32/28	23/14	6/6	1/7	2/1
Age at disease onset (median, range)	32 (14–72)	28 (14–62)	65 (35–72)	54.5 (43–64)	32 (26–40)
Time of diagnosis (median, range)	19 (3–540)	20 (9–540)	17 (3–370)	40 (5–120)	10 (7–40)
mRS at the peak of disease (median, range)	5 (4–5)	5 (4–5)	5 (4–5)	4 (4–5)	5 (5–5)
ICU admission (*n*, %)	24 (40)	20 (54)	3 (37.5)	0	1 (33)
Tumor (*n*, %)	14 (23.3)	5 (13.5)	9 (75)	0	0
Tumor type (*n*)		Ovarian teratoma (5)	SCLC(9)		
Abnormal MRI (*n*, %)	20 (33.3)	13 (35)	4 (33.3)	3 (37.5)	0
Abnormal CSF (*n*, %)	30 (50)	22 (59.4)	7 (58.3)	1 (12.5)	0
CSF protein [g/L (median, range)]	0.3 (0.1–1.39)	0.29 (0.1–1.39)	0.36 (0.21–0.59)	0.295 (0.21–0.63)	0.3 (0.2–0.45)
Elevated CSF protein (*n*, %)	11 (18.3)	8 (21.6)	2 (16.7)	1 (12.5)	0
CSF WCC [cells/L(median, range)]	10 (1–286)	16 (1–286)	13 (3–118)	3 (1–8)	6 (2–8)
Elevated CSF WCC (*n*, %)	25 (41.7)	19 (51.3)	6 (50)	0	0

mRS, modified Rankin scale; ICU, intensive care unit; MRI, magnetic resonance imaging; CSF, cerebrospinal fluid; WCC, white cell count; SCLC, small-cell lung cancer; NMDAR, N-methyl-d-aspartate receptor; CASPR2, contactin-associated protein-like 2; GABAbR, g-aminobutyric acid receptor B; LGI1, leucine-rich glioma-inactivated protein 1.

**Figure 3:**
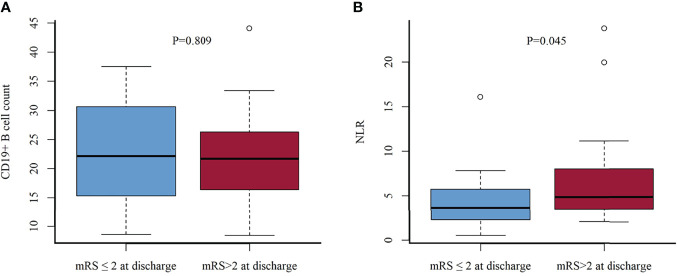
CD19^+^ B-cell count **(A)** and NLR **(B)** in functional independence group (mRS scores, ≤2) vs. nonindependence group (mRS scores, >2) at discharge. NLR, neutrophil-to-lymphocyte ratio.

The CD19^+^ B-cell count was similar between those patients who had reached functional independence at discharge from the hospital (mRS, ≤2) and those with nonfunctional independence (mRS, >2) (22.84 ± 8.61 vs 22.16 ± 7.98; *p* = 0.809) (**Figure 3A**). NLR was higher in patients without functional independence (range, 2.07–23.7; median, 4.87) than in patients with functional independence (range, 0.51–16.07; median, 3.67) (*p* = 0.045) (**Figure 3B**).

### Treatment Outcomes

No randomized controlled trials have yet been conducted to investigate standard immunotherapy protocols for AE. In our cohort, 60/60 (100%) patients received high-dose corticosteroids (500–1,000 mg) for 5 days, and IVIG was administered to 52/60 (86.7%) patients. Overall, 36/60 (60%) patients responded to first-line immunotherapy, and the median change in mRS score was 1 (range, 0–3). Compared to the other subgroups, the LGI1-AE subgroup exhibited greater mRS improvement, but the difference was not significant (**Table 2**) (*p* = 0.262). The median mRS after first-line therapy in the entire cohort was 4 (range, 1–6).

**Table 2:** Immunotherapy and follow-up of patients.

	Total cases	NMDAR	GABAbR	LGI1	CASPR2
First-line therapy					
Steroids (*n*, %)	60 (100)	37 (100)	12 (100)	8 (100)	3 (100)
IVIG (*n*, %)	52 (86.7)	36 (97)	9 (75)	4 (50)	3 (100)
Response to first-line therapy (*n*, %)	36 (60)	19 (51.3)	8 (66.7)	7 (87.5)	2 (66.7)
Δ mRS scores post-first-line therapy (median, range)	1 (0–3)	1 (0–2)	1 (0–3)	2 (0–3)	1 (0–2)
mRS score post-first-line therapy (median, range)	4 (1–6)	4 (2–6)	4 (2–5)	3 (1–4)	4 (3–5)
Second-line therapy (*n*, %)	26 (43.3)	20 (54.1)	2 (16.7)	3 (37.5)	1 (33.3)
Rituximab (*n*, %)	26 (43.3)	20 (54.1)	2 (16.7)	3 (37.5)	1 (33.3)
Cyclophosphamide (*n*, %)	1 (1.7)	1 (2.9)	0	0	0
Add on immunotherapy					
Bortezomib (*n*, %)	7 (11.7)	7 (18.9)	0	0	0
Long-term immunotherapy					
Mycophenolate mofetil [MMF (*n*, %)]	24 (40)	20 (54.1)	1 (8.3)	2 (25)	1 (33.3)
mRS score at discharge (median, range)	3 (2–6)	3 (2–6)	3 (2–5)	2 (2–2)	3 (2–3)
mRS score ≤2 at discharge (*n*, %)	28 (46.7)	16 (43.2)	3 (25)	8 (100)	1 (33.3)
mRS score at final follow-up (median, range)	1 (0–6)	1 (0–6)	6 (0–6)	1 (0–2)	2 (0–2)
mRS score ≤2 at final follow-up (*n*, %)	48 (80)	32 (86.5)	5 (41.7)	8 (100)	3 (100)
Relapse (*n*, %)	17 (28.3)	10 (27)	6 (50)	1 (12.5)	0
Mortality (*n*, %)	10 (16.7)	3 (8.1)	7 (58.3)	0	0

IVIG, intravenous immunoglobulins; mRS, modified Rankin scale; Δ mRS, changes in the mRS; NMDAR, N-methyl-d-aspartate receptor; CASPR2, contactin-associated protein-like 2; GABABR, g-aminobutyric acid receptor B; LGI1, leucine-rich glioma-inactivated protein 1.

Second-line immunotherapy was initiated 25 (range, 5–300) days after the definitive diagnosis. Rituximab, cyclophosphamide, or a combination of the two were implemented in 26/60 (43.3%) patients who showed no significant improvement to first-line therapy or experienced a definite clinical relapse. Rituximab (26 patients, 43.3%) was the most frequently applied second-line immunotherapy with two regimens (100 mg IV infusion once per week for 4 consecutive weeks or 600 mg IV infusion in 1 day). In total, 19/26 (73.1%) patients treated with rituximab showed significant improvement, and 7/26 (26.9%) patients pretreated with rituximab were still refractory and received further immunosuppressant drugs with bortezomib as an add-on therapy at a median time of 32 (range, 29–45) days after the last dose of rituximab. A total dose of 1.3 mg/m^2^ was administered subcutaneously on days 1, 4, 8, and 11 of the 21-day cycle. Each patient received a median of 1 (range, 1–3) cycle. Although 36/60 (60%) patients showed improvement after first-line immunotherapy, based on the severity of the initial attack and the risk of relapse, long-term immunosuppression (MMF) was administered in 24/60 (40%) patients. At discharge, the median mRS score was 3 (range 2–6), which was significantly lower than the score of 5 (range 4–5) at the peak of the disease (P < 0.001). Surprisingly, anti-LGI1 encephalitis patients typically showed substantial recovery, with none having a moderate or severe deficit at discharge.

**Figure 4:**
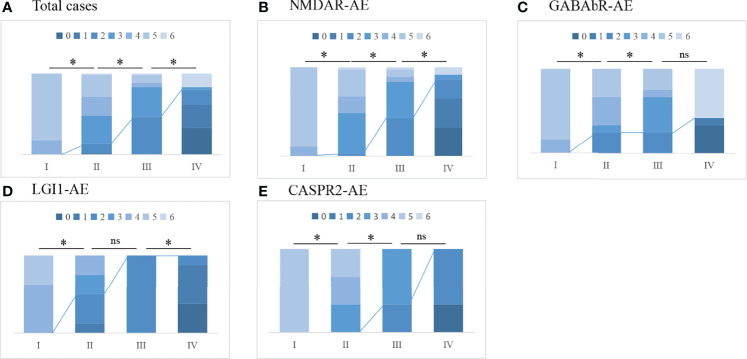
The change in mRS scores and the outcome of total cases **(A)** and different subtypes of AE **(B–E)**. I, maximal mRS at symptom onset; II, mRS post-first-line immunotherapy; III, mRS at discharge from hospital; IV, mRS at last follow-up. The line represents the change in mRS scores dividing favorable mRS scores (0–2) and unfavorable mRS scores (≥3); *p < 0.05. ns, not significant.

The mean duration of follow-up was 40 (range, 1–84) months. The distribution of mRS scores at the peak of the disease and the last follow-up improved significantly in patients with NMDAR-AE, LGI1-AE, and CASPR2-AE; no significant improvement was observed in patients in the GABAbR-AE group (**Figure 4**). Although patients with severe AE were severely affected at baseline (**Table 1**), at the final follow-up, 48/60 (80%) patients had achieved independent living (mRS score, ≤2) with a median mRS of 1 (0–6) (*p* < 0.01) (**Table 2**). Ten (16.7%) patients (three NMDAR-AE, seven GABAbR-AE) died of consequences associated with lung tumors, symptoms, and severe bacteremia.

Relapses occurred in 17/60 (28.3%) patients in our study at a median time of 10 months (2–36 months) after the initial episode (10 anti-NMDAR encephalitis cases, 6 GABAbR encephalitis cases, and 1 LGI1 encephalitis). Of the relapsed cases, 9/17 (52.9%) were treated with second-line immunotherapy or long-term immunotherapy after the initial attack. One patient with NMDAR-AE relapse had an ovarian teratoma at disease onset, and six patients with GABAbR-AE relapse had small-cell lung cancer. Three of 17 (17.6%) patients experienced further relapses (range, 2–7). All relapsed patients underwent reinitiation of first-line immunotherapy, six patients subsequently received long-term MMF, and 11 patients were treated with rituximab.

### Predictors of Treatment Efficiency and Prognosis

Univariate logistic regression analysis indicated that younger age (*p* = 0.045), nontumor status (*p* = 0.003), nonpulmonary infection complications (*p* = 0.007), lower NLR levels (*p* = 0.022), and response to first-line treatment (*p* = 0.005) were associated with good outcomes at the final follow-up. A high CD19^+^ B-cell count corresponded with failure of first-line treatment (OR, 1.109; 95% CI [1.013–1.24]; *p* = 0.04). Sex, CSF protein, and early diagnosis were not related to any of our outcomes (**Table 3**). Multivariable logistic regression analysis confirmed that patients with a high CD19^+^ B-cell count exhibited an OR of 1.197 (95% CI [1.043–1.496]) for predicting failure of first-line treatment at a statistically significant level (*p* = 0.041). Lower NLR levels were more likely to have good functional outcomes at final follow-up for severe AE (OR, 0.686; 95% CI [0.472–0.884]; *p* = 0.015). Tumors corresponded with increased odds of relapse (OR, 29.506; 95% CI [2.79–757.342]; *p* = 0.014) and mortality (OR, 8.034, 95% CI [1.388–58.033]; *p* = 0.024) (**Table 4**).

**Table 3:** Univariate logistic regression analysis for all severe AE patients.

Variables [OR ([95% CI); *p*-value]	mRS ≤2 at discharge	mRS ≤2 at final follow-up	ICU admission	Failure of first-line treatment	Mortality	Relapse
Age	0.996 [0.968–1.025]; *p* = 0.802	**0.964 [0.928–0.998]; *p*** **=** **0.045**	0.975 [0.944–1.004]; *p* = 0.104	0.976 [0.946–1.005]; *p* = 0.12	**1.054 [1.014–1.102]; *p*** **=** **0.012**	0.991 [0.958–1.023]; *p* = 0.588
Gender	1.686 [0.609–4.776]; *p* = 0.317	1.533 [0.445–5.724]; *p* = 0.504	0.946 [0.332–2.672]; *p* = 0.916	0.946 [0.332–2.672]; *p* = 0.916	0.595 [0.141–2.233]; *p* = 0.451	0.6 [0.177–1.904]; *p* = 0.393
Tumor	**0.147 [0.021–0.623]; *p* = 0.02**	**0.125 [0.029–0.49]; *p* = 0.003**	2.059 [0.592–7.384]; *p* = 0.255	0.921 [0.246–3.199]; *p* = 0.898	**12.542 [2.958–62.048]**; ***p* = 0.001**	3.171 [0.854–11.819]; *p* = 0.081
Pulmonary infection complications	**0.087 [0.021**–**0.291]**; ***p* < 0.01**	**0.141 [0.028**–**0.535]**; ***p* = 0.007**	**391 [49.681**–**9387.565]**; ***p* < 0.01**	**8.500 [2.736**–**29.527]**; ***p* < 0.01**	**5.02 [1.27**–**25.275]**; ***p* = 0.029**	0.365 [0.091–1.232]; *p* = 0.122
Early diagnosis	0.765 [0.274–2.113]; *p* = 0.605	0.821 [0.232–2.834]; *p* = 0.754	1.750 [0.621–5.080]; *p* = 0.294	2.333 [0.821–6.933]; *p* = 0.117	1.25 [0.334–4.86]; *p* = 0.739	1.408 [0.446–4.588]; *p* = 0.56
CSF WCC	0.986 [0.966–1.001]; *p* = 0.115	0.993 [0.979–1.008]; *p* = 0.343	**1.018 [1.003–1.038]; *p* = 0.038**	1.003 [0.989–1.016]; *p* = 0.668	1.007 [0.992–1.022]; *p* = 0.315	0.994 [0.971–1.009]; *p* = 0.488
CSF protein	2.851 [0.077–124.592]; *p* = 0.566	0.838 [0.014–88.474]; *p* = 0.935	1.201 [0.029–46.085]; *p* = 0.92	0.167 [0.002–6.715]; *p* = 0.367	0.772 [0.004–58.939]; *p* = 0.913	0.289 [0.002–18.328]; *p* = 0.588
CD19^+^ B-cell count	1.011 [0.930–1.100]; *p* = 0.800	1.128 [0.954–1.421]; *p* = 0.219	0.953 [0.867–1.037]; *p* = 0.283	**1.109 [1.013–1.24]; *p* = 0.04**	0.814 [0.556–1.026]; *p* = 0.162	0.947 [0.857–1.034]; *p* = 0.248
NLR	0.856 [0.693–0.997]; *p* = 0.089	**0.823 [0.677–0.953]; *p* = 0.022**	1.063 [0.937–1.225]; *p* = 0.346	1.108 [0.974–1.297]; *p* = 0.146	1.105 [0.960–1.280]; *p* = 0.150	0.970 [0.813–1.110]; *p* = 0.686
Failure of first-line treatment	**0.149 [0.041–0.465]; *p* = 0.002**	**0.127 [0.026–0.487]; *p* = 0.005**	**7.00[2.290–23.562]; *p* = 0.001**	–	**5.5 [1.387–27.784]; *p* = 0.022**	0.598 [0.165–1.947]; *p* = 0.407

Univariate logistic regression analyses was performed to determine correlations between covariates (including NLR, CD19^+^ B-cell count) and the outcomes(mRS, ICU admission, failure of first-line treatment, mortality, relapse). OR, 95% CI, and their respective p-values are shown for all correlations. Significant values (p < 0.05) are highlighted (in bold). Lower NLR level was associated with good outcome at final follow-up [OR, 0.823; 95% CI (0.677–0.953); p = 0.022]. High CD19^+^ B-cell count corresponded with failure of first-line treatment [OR, 1.109; 95% CI (1.013–1.24); p = 0.04]. mRS, modified Rankin scale; CSF, cerebrospinal fluid; WCC, white cell count; NLR, neutrophil-to-lymphocyte ratio; ICU, intensive care unit; OR, odds ratios; CI, confidence intervals.

**Table 4:** Multivariable logistic regression analysis for all severe AE patients.

Variables [OR (95% CI); *p*-value]	mRS ≤2 at discharge	mRS ≤2 at final follow-up	ICU admission	Failure of first-line treatment	Mortality	Relapse
Age	0.984 [0.936–1.031]; *p* = 0.51	0.895 [0.801–0.963]; *p* = 0.013	0.895 [0.726–0.982]; *p* = 0.094	1.003 [0.949–1.065]; *p* = 0.914	**1.083 [1.021–1.174]; *p* = 0.02**	0.956 [0.905–1.003]; *p* = 0.082
Gender	2.575 [0.565–13.698]; *p* = 0.236	2.797 [0.277–47.809]; *p* = 0.405	–	–	–	1.905 [0.346–12.874]; *p* = 0.473
Tumor	0.243 [0.01–2.53]; *p* = 0.281	0.426 [0.048–3.443]; *p* = 0.42	–	1.517 [0.083–32.847]; *p* = 0.772	**8.034 [1.388–58.033]; *p* = 0.024**	**29.506 [2.79–757.342]; *p* = 0.014**
Pulmonary infection complications	**0.082 [0.01–0.437]; *p* = 0.007**	**0.014 [0–0.196]; *p* = 0.008**	**6895.308 [100.529–470,891,290.905]; *p* = 0.008**	**20.15 [2.054–522.743]; *p* = 0.028**	**16.376 [1.68–387.747]; *p* = 0.035**	**0.069 [0.004–0.553]; *p* = 0.03**
CSF WCC	1.003 [0.981–1.022]; *p* = 0.755	1.015 [0.994–1.045]; *p* = 0.217	–	0.995 [0.97–1.021]; *p* = 0.692	1.001 [0.981–1.018]; *p* = 0.912	0.996 [0.966–1.021]; *p* = 0.772
CD19^+^ B-cell count	–	–	–	**1.197 [1.043–1.496]; *p* = 0.041**	–	–
NLR	0.835 [0.624–1.03]; *p* = 0.146	**0.686 [0.472–0.884]; *p* = 0.015**	0.851 [0.548–1.279]; *p* = 0.357	–	–	0.846 [0.611–1.082]; *p* = 0.252
Failure of first-line treatment	–	–	1.14 [0.044–17.424]; *p* = 0.925	–	–	–

Variables with statistical significance in the univariate logistic regression analysis and clinically relevant variables were included in multivariable logistic regression models. OR, 95% CI, and their respective p-values are shown for all correlations. Significant values (p < 0.05) are highlighted (in bold). High CD19^+^ B-cell count has exhibited an OR of 1.197 (95% CI = 1.043–1.496) for predicting failure of first-line treatment at a statistically significant level (p = 0.041). Lower NLR levels were more likely to have good functional outcome at final follow-up of severe AE [OR, 0.686; 95% CI (0.472–0.884); p = 0.015]. mRS, modified Rankin scale; CSF, cerebrospinal fluid; WCC, white cell count; NLR, neutrophil-to-lymphocyte ratio; ICU, intensive care unit; OR, odds ratios; CI, confidence intervals.

**Figure 5:**
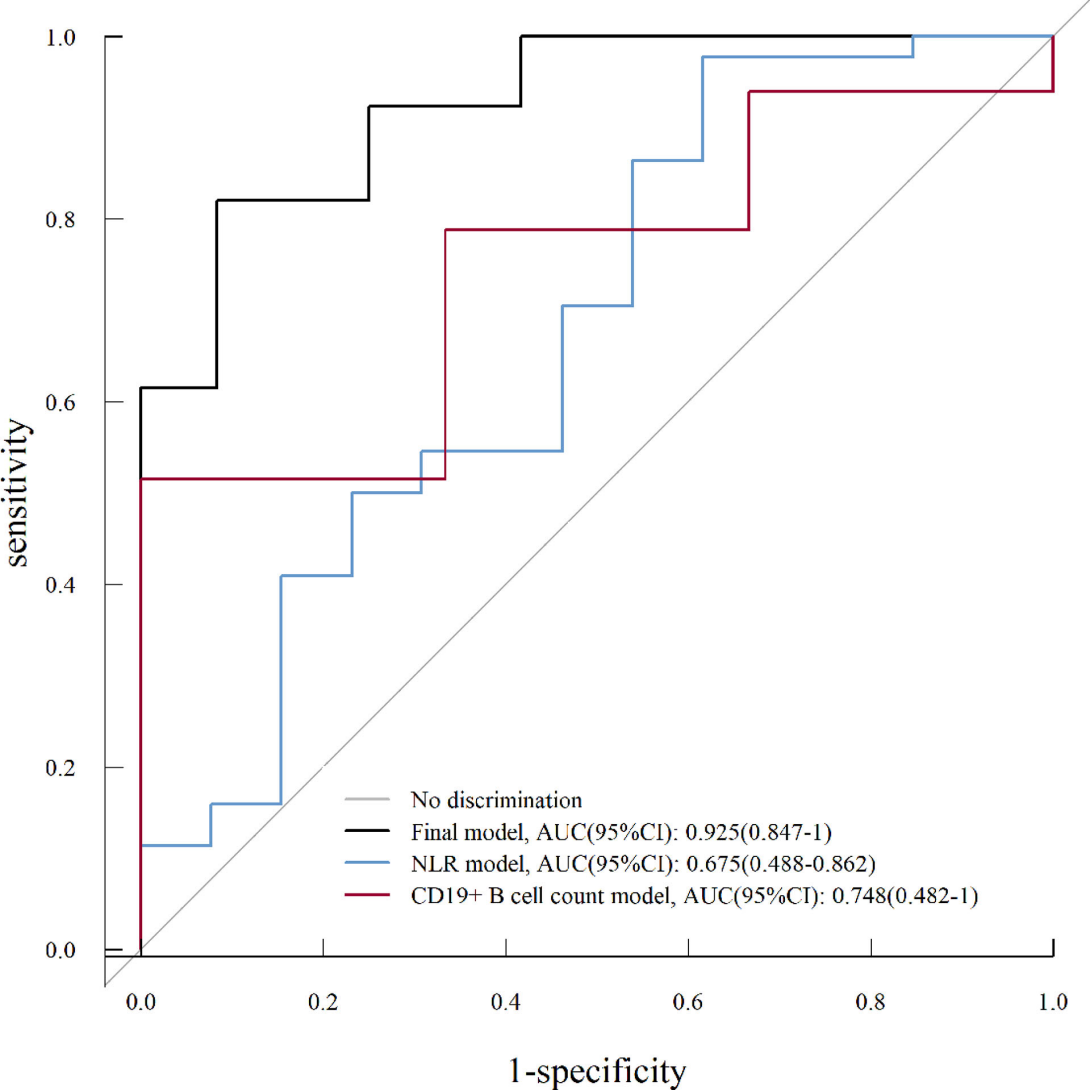
Receiver operating characteristic (ROC) curve analysis for a univariate analysis (NLR model, CD19^+^ B-cell count model) and multivariate analysis (final model) on good prognosis (mRS score, ≤2) of severe AE. NLR, neutrophil-to-lymphocyte ratio; AUC, area under the curve; CI, confidence interval.

The ROC curve analysis was performed to evaluate the predictive value for a good outcome at the final follow-up using the full multivariate model (final model) and univariate model (NLR model, CD19^+^ B-cell count model). As shown in **Figure 5**, the full multivariate model demonstrated a good predictive value [AUC = 0.925; 95% CI (0.847–1)] compared to the univariate model. Based on the ROC curve, the optimal cutoff values of NLR and CD19^+^ B-cell count to predict good outcomes were 10.19 (sensitivity, 0.977; specificity, 0.384) and 22.33 (sensitivity, 0.515; specificity, 1), respectively (**Figure 5**).

## Discussion

In this retrospective analysis, we identified several novel findings. First, this is the most detailed description of clinical features in patients with severe AE to date, and long-term outcomes in the overall cohort were favorable. Second, we showed that a lower dosage of rituximab is the most frequently applied second-line immunotherapy used in 43.3% of all patients with severe AE. We also observed a clinical benefit and provided preliminary evidence that a lower dosage of rituximab may be as effective as standard doses for treating severe AE with good tolerance and less financial burden. Finally, we found that CD19^+^ B-cell count and NLR can help predict the response to treatment and prognosis, respectively, and could thus be valuable in guiding clinicians to offer aggressive rescue immunotherapy.

In our cohort, severe AE cases mostly comprised anti-NMDAR encephalitis. The sex ratio and age distribution among those with anti-NMDAR encephalitis were similar to those of previous studies, showing a higher frequency in women (14–16). However, the sex disparity in the rest of the subtypes was not in concordance with the results of prior studies, which reported male predominance in LGI1, GABAbR, and CASPR2 encephalitis (17). In our cohort, cerebellar ataxia and brainstem encephalitis were uncommon manifestations that occurred in patients with NMDAR-AE and GABAbR-AE. When altered consciousness is accompanied by these atypical symptoms, Bickerstaff encephalitis, which is characterized by a typical picture of cranial nerve involvement and consciousness alterations, should be excluded (18). Data regarding the prevalence of tumor association was also confirmed in our study; the occurrence of an underlying teratoma in female patients was lower (13.5%) compared to those reported in previous publications (19.5%–58%) (16, 17, 19). The reasons for heterogeneity may be explained by the inclusion criteria, sample sizes, or other factors, including genetic background and epidemiology. In GABAbR encephalitis, the tumor type was small-cell lung cancer, occurring in 75% of anti-GABABR-positive patients, which is in accordance with the findings of Hayden et al. (20). In tumor-associated AE, surgical treatment or radiation/chemotherapy should be initiated as soon as possible to relieve the symptoms and allow a more favorable long-term outcome. In the study by Lee et al. (19), nine teratomas were not detected in the initial workup but by a follow-up pelvic MRI, resulting in delayed removal of the teratoma, which suggests that the extent of tumor screening and regular tumor screening should be taken into consideration.

Inflammatory changes in the CSF were noted in 30 (50%) of the patients. We found that patients in the NMDAR-AE and GABAbR-AE groups were more likely to develop CSF pleocytosis than the other subtypes of the cohort; this result is similar to those of other studies (20, 21). Previous studies have found an association between CSF changes and worse outcomes (22). In the current cohort, we also confirmed that abnormal CSF white cell counts increased the odds of ICU admission.

The NLR has previously been proposed as an indicator of systemic inflammation. A high NLR implies overwhelmed inflammation or imbalanced innate and adaptive immunity, which are frequently used to predict outcomes (23). Our results showed that NLR was higher in patients without functional independence at discharge, while those with lower NLR levels were more likely to have good functional outcomes at final follow-up (OR, 0.686; 95% CI [0.472–0.884]; *p* = 0.015). This was in line with earlier reports that noted that the percentage of patients who exhibited severe disease increased significantly in the higher NLR subgroup, and a high NLR was associated with higher odds of first-line treatment failure in AE (24, 25). In addition to NLR, evaluation of the peripheral CD19^+^ B-cell count revealed that a high CD19^+^ B-cell count is a predictor of first-line treatment failure. B cells are the major effector cells in AE through antibody production and proinflammatory cytokine production. However, the effects of first-line immunotherapy, such as corticosteroids, on B-lymphocytes are limited (26). This indicates that drugs targeting B lymphocytes are required.

Decisions regarding immunotherapy initiation were based on clinical symptoms. Our data confirmed that 36/60 (60%) patients responded to first-line immunotherapy, and anti-LGI1 encephalitis was associated with faster recovery, possibly due to low-affinity IgG4 antibodies (27). Recently, a study of the largest Chinese anti-NMDAR encephalitis cohort concluded that repeated first-line immunotherapy, involving mostly a combination of steroids and IVIG, can achieve favorable clinical outcomes (16). Notably, Zhang et al. reported that therapeutic plasma exchange might be an effective rescue therapy associated with rapid functional improvement in patients with severe steroid/IVIG-refractory antibody-associated AE (28).

In addition, early initiation of second-line immunotherapy with rituximab has been shown to result in a more favorable prognosis (11, 12). In the meta-analysis of Nepal et al. (11), good outcomes at last follow-up were noted in 71.8% of patients following rituximab therapy, with a mean mRS score decrease of 2.67, and relapse occurred in only 17.5% of patients with an acceptable toxicity profile. Similarly, in a study by Thaler et al. (12), early and short-term rituximab therapy was shown to be an effective and safe treatment option in most patients with NMDAR-, LGI1-, and CASPR2-AE. These study outcomes were consistent with the therapeutic outcomes for rituximab in AE. In our study, in terms of rituximab dose, we used reduced-dose rituximab, considering rituximab’s off-label use for AE in China and the cost of hospitalization. The median time lag from definite diagnosis of the disease to rituximab administration in our study was 25 (range, 5–300) days. As rituximab is used as a second-line drug, the delay in initiation of rituximab therapy may affect the outcomes. We found that 19/26 (73.1%) patients treated with rituximab showed a significant improvement, and 7/26 (26.9%) patients were still refractory, consistent with Titulaer et al. (4, 11), while the mechanism of rituximab-resistant AE remains undefined. In a novel immune-mediated model of anti-NMDAR encephalitis provided by Wagnon et al. (8), the differentiation of B cells into plasma cells coincided with an increase in protein concentration and the detection of anti-NMDAR IgG in the CSF. In anti-NMDAR encephalitis brains, antibody-secreting cells (ASCs) reside in perivascular, interstitial, and Virchow–Robin spaces, which may be the main source of continuously synthesized Ig (20). This suggests that these plasma cells are responsible for the production of anti-NMDAR autoantibodies that may contribute to disease progression. Previous studies have demonstrated the rescuing effects of proteasome inhibitor bortezomib in patients unresponsive to rituximab, depleting extra-CNS ASCs in a targeted manner (9, 13). In our cohort, seven patients received bortezomib treatment, six showed clinical improvement, and one patient died due to serious complications.

Therapeutic recommendations related to the long-term management of AE are influenced by multiple factors: (1) the presence and type of neuronal autoantibodies and their relevance to the patient’s presentation, (2) relapse rates in different AE subtypes, and (3) severity of the initial attack and individual risks related to immunosuppression. Of note, overlapping with oral corticosteroids is needed for 3–6 months when using MMF owing to its delayed onset of action (29). Moreover, vigilant management of airway complications, especially pulmonary infection and monoclonal antibody infusion-related reactions, is required.

In our cohort, the relapse rate was 28.3%, compared with rates of 10%–35% calculated in previous studies (4, 17, 30). Tumors corresponded with increased odds of relapse (OR, 29.506; 95% CI [2.79–757.342]; *p* = 0.014). The high relapse rate may be due to the severity of the disease, the detection of tumors, and the fact that some patients were not treated with second-line immunotherapies or long-term immunotherapy after the initial attack. Patients who experience a definite relapse should be treated with the same first-line treatment scheme as at the first clinical presentation; for long-term immunosuppression, rituximab is the most popular choice, chosen by 46% of responders for relapsing AE (29).

Our treatment regimens showed promising outcomes as more than 46.7% of patients had an mRS of ≤2 at discharge, and 48/60 (80%) patients had achieved independent living (mRS score, ≤2) at final follow-up, which may be due to the early and high-frequency (43.3%) application of rituximab and aggressive administration of bortezomib. This study also provides preliminary evidence that lower doses of rituximab may be as effective as the standard doses to treat severe AE.

This study had some limitations. First, the retrospective collection and analysis of clinical information and the lack of a control cohort resulted in the heterogeneity of rituximab treatment regimens. Prolonged monitoring is required to assess the long-term efficacy of rituximab in treating severe AE. The second constraint is the relatively small sample size of the cohort made up of different subtypes of severe AE, which may introduce bias in this study. Furthermore, the mRS is a scale developed to measure global disability, and a novel clinical scale, such as the clinical assessment scale in autoimmune encephalitis (CASE), should be applied to evaluate the severity in patients with diverse AE syndromes (31). Prospective multicenter studies are required to address this question.

In summary, we showed the clinical characteristics of severe AE and the predictive value of peripheral immune cells for treatment response and prognosis. We present evidence of the efficacy of early lower dosage of rituximab treatment in severe AE and suggest that short-term therapy could be a viable treatment option; bortezomib can be used as rescue immunotherapy in rituximab-resistant patients. Future studies are needed to investigate new therapeutic strategies, such as IL-6 receptor blockers, which may interfere with the pathologic activation of B cells (tocilizumab) and anti-CD19 agents (inebilizumab). More *in vitro* and *in vivo* studies are needed to improve our understanding of the molecular mechanism of severe AE.

## Data Availability Statement

The original contributions presented in the study are included in the article/supplementary material. Further inquiries can be directed to the corresponding author.

## Ethics Statement

The studies involving human participants were reviewed and approved by the ethics institutional review board of Shandong Provincial Hospital. Written informed consent to participate in this study was provided by the participants’ legal guardian/next of kin.

## Author Contributions

BW collected the data and drafted the manuscript. CW and JF analyzed the data. MH rechecked the data and revised the manuscript. SG designed and conceptualized the study, interpreted the data, and revised the manuscript. All authors contributed to the article and approved the submitted version.

## Funding

This work was supported by the National Natural Science Foundation of China (Grant No. NSFC82072079).

## Conflict of Interest

The authors declare that the research was conducted in the absence of any commercial or financial relationships that could be construed as a potential conflict of interest.

## Publisher’s Note

All claims expressed in this article are solely those of the authors and do not necessarily represent those of their affiliated organizations, or those of the publisher, the editors and the reviewers. Any product that may be evaluated in this article, or claim that may be made by its manufacturer, is not guaranteed or endorsed by the publisher.
